# The Versatile Mutational Resistome of *Pseudomonas aeruginosa*

**DOI:** 10.3389/fmicb.2018.00685

**Published:** 2018-04-06

**Authors:** Carla López-Causapé, Gabriel Cabot, Ester del Barrio-Tofiño, Antonio Oliver

**Affiliations:** Servicio de Microbiología y Unidad de Investigación, Hospital Universitari Son Espases, Institut d’Investigació Sanitaria Illes Balears, Palma de Mallorca, Spain

**Keywords:** antibiotic resistance, resistome, *Pseudomonas aeruginosa*, multidrug resistance, evolution, resistance development, mutation

## Abstract

One of the most striking features of *Pseudomonas aeruginosa* is its outstanding capacity for developing antimicrobial resistance to nearly all available antipseudomonal agents through the selection of chromosomal mutations, leading to the failure of the treatment of severe hospital-acquired or chronic infections. Recent whole-genome sequencing (WGS) data obtained from *in vitro* assays on the evolution of antibiotic resistance, *in vivo* monitoring of antimicrobial resistance development, analysis of sequential cystic fibrosis isolates, and characterization of widespread epidemic high-risk clones have provided new insights into the evolutionary dynamics and mechanisms of *P. aeruginosa* antibiotic resistance, thus motivating this review. Indeed, the analysis of the WGS mutational resistome has proven to be useful for understanding the evolutionary dynamics of classical resistance pathways and to describe new mechanisms for the majority of antipseudomonal classes, including β-lactams, aminoglycosides, fluoroquinolones, or polymixins. Beyond addressing a relevant scientific question, the analysis of the *P. aeruginosa* mutational resistome is expected to be useful, together with the analysis of the horizontally-acquired resistance determinants, for establishing the antibiotic resistance genotype, which should correlate with the antibiotic resistance phenotype and as such, it should be useful for the design of therapeutic strategies and for monitoring the efficacy of administered antibiotic treatments. However, further experimental research and new bioinformatics tools are still needed to overcome the interpretation limitations imposed by the complex interactions (including those leading to collateral resistance or susceptibility) between the 100s of genes involved in the mutational resistome, as well as the frequent difficulties for differentiating relevant mutations from simple natural polymorphisms.

## Introduction

*Pseudomonas aeruginosa* is one of the most frequent and severe causes of hospital-acquired infections, particularly affecting immunocompromised (especially neutropenic) and Intensive Care Unit (ICU) patients. Indeed, *P. aeruginosa* is the first cause of ventilator associated pneumonia (VAP) and burn wound infections, both associated with a very high mortality rate (Vincent, 2003; Bassetti et al., 2012). Likewise, *P. aeruginosa* is the most frequent driver of chronic respiratory infections in cystic fibrosis (CF) patients or other chronic underlying diseases (Döring et al., 2011).

One of the most striking features of *P. aeruginosa* is its outstanding capacity for developing antimicrobial resistance to nearly all available antipseudomonal agents through the selection of chromosomal mutations. Indeed, treatment failure caused by the development of antimicrobial resistance is a too frequent outcome of *P. aeruginosa* infections. The problem of mutation-mediated antibiotic resistance is further amplified in the chronic infection setting, due to the very high prevalence of hypermutable strains, showing greatly enhanced spontaneous mutation rates caused by defective DNA repair or error avoidance systems (Oliver et al., 2000; Maciá et al., 2005).

Beyond the obvious negative impact of resistance development for the treated patient, the accumulation of several of these chromosomal mutations leads to the emergence of multidrug resistant (MDR), extensively drug-resistant (XDR) or even pan-antibiotic-resistant (PDR) strains, which can be responsible for notable epidemics in the hospital setting (Deplano et al., 2005; Suarez et al., 2011). Moreover, recent studies have evidenced the existence of MDR/XDR global clones disseminated in different hospitals worldwide, and for that reason they have been denominated high-risk clones (Woodford et al., 2011; Oliver et al., 2015). Although high-risk clones are frequently associated with transferable antimicrobial resistance determinants, they also typically show a wide range of chromosomal mutations playing a major role in the resistance phenotype (Oliver et al., 2015). Likewise, recent reports have evidenced the interpatient spread of antimicrobial resistance mutations linked to the transmission of epidemic CF strains (López-Causapé et al., 2017).

Along with growing information from mechanistic studies on chromosomal resistance mechanisms and their complex regulatory pathways, involved in adaptive resistance (Lister et al., 2009; Muller et al., 2011; Skiada et al., 2011; Juan et al., 2017), the introduction of whole-genome sequencing (WGS) approaches is shaping up a new dimension for the mutational resistance landscape. The term resistome was first used to account for the set of primary antibiotic resistance genes that could be eventually transferred to human pathogens (D’Costa et al., 2006). Soon after the concept of intrinsic resistome was introduced to include all chromosomal genes that are involved in intrinsic resistance, and whose presence in strains of a bacterial species is independent of previous antibiotic exposure and is not due to horizontal gene transfer (HGT) (Fajardo et al., 2008). Finally, the term mutational resistome was more recently implemented to account for the set of mutations involved in the modulation of antibiotic resistance levels in the absence of HGT (Cabot et al., 2016b; López-Causapé et al., 2017). Recent WGS data obtained from *in vitro* assays on the evolution of antibiotic resistance, *in vivo* monitoring of antimicrobial resistance development, analysis of sequential CF isolates, and characterization of wide spread epidemic high-risk clones provide new insights into the evolutionary dynamics and mechanisms of *P. aeruginosa* antibiotic resistance (Cabot et al., 2016a; Feng et al., 2016; Del Barrio-Tofiño et al., 2017; Jaillard et al., 2017; López-Causapé et al., 2017). Indeed, the analysis of WGS mutational resistomes has proven to be useful for understanding the evolutionary dynamics of classical resistance mechanisms and to depict new ones for the majority of antimicrobial classes, including β-lactams, aminoglycosides, fluoroquinolones, polymixins and others, as reviewed in the following sections. **Table 1** summarizes the main genes and mutations known to increase resistance levels and therefore shaping up the *P. aeruginosa* mutational resistome.

**Table 1:** Main genes and mutations known to be involved in increased *P. aeruginosa* antibiotic resistance.

Gene	Resistance mechanisms/altered target	Antibiotics affected^a^	Type of mutation	Relevant examples of gain-of-function mutations	Reference
*gyrA*	DNA gyrase	FQ	Gain-of-function	G81D, T83A, T83I, Y86N, D87G, D87N, D87Y, Q106L	Bruchmann et al., 2013; Kos et al., 2015; Cabot et al., 2016b; Del Barrio-Tofiño et al., 2017; López-Causapé et al., 2017
*gyrB*	DNA gyrase	FQ	Gain-of-function	S466F, S466Y, Q467R, E468D	Bruchmann et al., 2013; Kos et al., 2015; Del Barrio-Tofiño et al., 2017; López-Causapé et al., 2017
*parC*	DNA topoisomerase IV	FQ	Gain-of-function	S87L, S87W	Bruchmann et al., 2013; Kos et al., 2015; Cabot et al., 2016b; Del Barrio-Tofiño et al., 2017
*parE*	DNA topoisomerase IV	FQ	Gain-of-function	S457G, S457T, E459D, E459K	Bruchmann et al., 2013; Kos et al., 2015; Del Barrio-Tofiño et al., 2017; López-Causapé et al., 2017
*pmrA*	Lipopolysaccharide (lipid A)	COL	Gain-of-function	L157Q	Lee and Ko, 2014
*pmrB*	Lipopolysaccharide (lipid A)	COL	Gain-of-function	L14P, A54V, R79H, R135Q, A247T, A248T, A248V, R259H, M292I, M292T	Barrow and Kwon, 2009; Moskowitz et al., 2012
*phoQ*	Lipopolysaccharide (lipid A)	COL	Loss-of-function		
*parR*	Lipopolysaccharide (lipid A)	COL	Gain-of-function	M59I, E156K	Muller et al., 2011; Guénard et al., 2014
	OprD downregulation	IMP, MER			
	MexEF-OprN hyperproduction	FQ			
	MexXY-OprM hyperproduction	FQ, AMG, CEF			
*parS*	Lipopolysaccharide (lipid A)	COL	Gain-of-function	L14Q, V101M, L137P, A138T, A168V Q232E, G361R	Muller et al., 2011; Fournier et al., 2013; Guénard et al., 2014
	OprD downregulation	IMP, MER			
	MexEF-OprN hyperproduction	FQ			
	MexXY-OprM hyperproduction	FQ, AMG, CEF			
*cprS*	Lipopolysaccharide (lipid A)	COL	Gain-of-function	R241C	Gutu et al., 2013
*colR*	Lipopolysaccharide (lipid A)	COL	Gain-of-function	D32N	Gutu et al., 2013
*colS*	Lipopolysaccharide (lipid A)	COL	Gain-of-function	A106V	Gutu et al., 2013
*mexR*	MexAB-OprM hyperproduction	FQ, CAZ, CEF, PPT, MER, CAZ/AVI	Loss-of-function		
*nalC*	MexAB-OprM hyperproduction	FQ, CAZ, CEF, PPT, MER, CAZ/AVI	Loss-of-function		
*nalD*	MexAB-OprM hyperproduction	FQ, CAZ, CEF, PPT, MER, CAZ/AVI	Loss-of-function		
*nfxB*	MexCD-OprJ hyperproduction	FQ, CEF	Loss-of-function		
*mexS*	MexEF-OprN hyperproduction	FQ	Loss-of-function		
	OprD downregulation	IMP, MER			
*mexT*	MexEF-OprN hyperproduction	FQ	Gain-of-function	G257S, G257A	Juarez et al., 2018
	OprD downregulation	IMP, MER			
*cmrA*	MexEF-OprN hyperproduction	MER, FQ	Gain-of-function	A68V, L89Q, H204L, N214K	Juarez et al., 2017
*mvaT*	MexEF-OprN hyperproduction	FQ	Loss-of-function		
*PA3271*	MexEF-OprN hyperproduction	FQ	Loss-of-function		
*mexZ*	MexXY-OprM hyperproduction	FQ, AMG, CEF	Loss-of-function		
PA5471.1	MexXY-OprM hyperproduction	FQ, AMG, CEF	Loss-of-function		
*amgS*	MexXY-OprM hyperproduction	FQ, AMG, CEF	Gain-of-function	V121G, R182C	Lau et al., 2015
*oprD*	OprD inactivation	IMP, MER	Loss-of-function		
*ampC*	AmpC structural modification	CAZ/AVI, C/T	Gain-of-function	T96I, G183D, E247K	Cabot et al., 2014; Fraile-Ribot et al., 2017a
*ampD*	AmpC hyperproduction	CAZ, CEF, PPT	Loss-of-function		
*ampDh2*	AmpC hyperproduction	CAZ, CEF, PPT	Loss-of-function		
*ampDh3*	AmpC hyperproduction	CAZ, CEF, PPT	Loss-of-function		
*ampR*	AmpC hyperproduction	CAZ, CEF, PPT	Gain-of-function	D135N, G154R	Bagge et al., 2002; Cabot et al., 2016b
*dacB*	AmpC hyperproduction	CAZ, CEF, PPT	Loss-of-function		
*ftsI*	Penicillin-binding-protein 3 (PBP3)	CAZ, CEF, PPT, MER, CAZ/AVI, C/T	Gain-of-function	R504C, R504H, P527T, F533L	Diaz Caballero et al., 2015; Cabot et al., 2016a,b; Del Barrio-Tofiño et al., 2017; López-Causapé et al., 2017
*fusA1*	Elongation factor G	AMG	Gain-of-function	I61M, V93A, E100G, K504E, Y552C, P554L, A555E, N592I, P618L, T671A, T671I	Feng et al., 2016; Bolard et al., 2017; Del Barrio-Tofiño et al., 2017; López-Causapé et al., 2017, 2018
*glpT*	Transporter protein GlpT	FOS	Loss-of-function		
*rpoB*	RNA polymerase β-chain	RIF	Gain-of-function	S517F, Q518R, Q518L, D521G, H531Y, H531L, S536F, L538I, S579F, S579Y, N629S, D636Y	Jatsenko et al., 2010

^a^*FQs, fluoroquinolones; COL, colistin; AMGs, aminoglycosides; CAZ, ceftazidime; CEF, cefepime; PPT, piperacillin-tazobactam; IMP, imipenem; MER, meropenem; CAZ/AVI, ceftazidime/avibactam; C/T, ceftolozane/tazobactam; FOS, fosfomycin; RIF, rifampicin*.

## β-Lactam Mutational Resistome

The most frequent mutation-driven β-lactam resistance mechanism is likely the overproduction of the chromosomal cephalosporinase AmpC, involving a wide range of genes belonging to complex regulatory pathways of cell-wall recycling (Juan et al., 2017). Among them, the mutational inactivation of *dacB*, encoding the non-essential penicillin-binding protein (PBP) PBP4, and *ampD*, encoding a *N*-acetyl-muramyl-L-alanine amidase have been found to be the most frequent cause of *ampC* derepression and β-lactam resistance (Juan et al., 2005; Moya et al., 2009). The inactivation of PBP4 has also been shown to activate the BlrAB/CreBC regulatory system, further increasing resistance levels (Moya et al., 2009). Additionally, specific point mutations leading to a conformation change in the transcriptional regulator AmpR, causing *ampC* upregulation and β-lactam resistance, have been noted among clinical strains. They include the D135N mutation, described in several species besides *P. aeruginosa*, including *Stenotrophomonas maltophilia*, *Citrobacter freundii*, or *Enterobacter cloacae* (Juan et al., 2017) or the R154H mutation, linked to the widespread MDR/XDR ST175 high-risk clone. Mutation of many other genes, including those encoding other amidases (AmpDh2 and AmpDh3), other PBPs (such as PBP5 and PBP7), lytic transglycosylases (such as SltB1 and mltB), MPL (UDP-*N*-acetylmuramate:Lalanyl-γ-D-glutamyl-meso-diaminopimelate ligase), or NuoN (NADH dehydrogenase I chain N) have been shown to enhance *ampC* expression, either alone or combined with other mutations, although their impact on β-lactam resistance among clinical strains still needs to be further analyzed (Juan et al., 2017).

In addition to *ampC* overexpression, recent studies have revealed that β-lactam resistance development, including the novel combinations of β-lactam-β-lactamase inhibitors ceftolozane/tazobactam and ceftazidime/avibactam, may result from mutations leading to the structural modification of AmpC (Cabot et al., 2014; Lahiri et al., 2014; Fraile-Ribot et al., 2017a; Haidar et al., 2017). Likewise, recent studies identified diverse AmpC variants associated with high-level cephalosporin resistance, including ceftolozane/tazobactam and ceftazidime/avibactam, in a small proportion (around 1%) of clinical *P. aeruginosa* isolates (Berrazeg et al., 2015). Over 200 Pseudomonas Derived Cephalosporinase (PDC) variants have been described so far, including those associated with enhanced ceftolozane/tazobactam and ceftazidime/avibactam resistance (**Table 1**). An update database of PDC variants is maintained in our laboratory and is freely available at https://arpbigidisba.com. Additionally, resistance development to ceftolozane/tazobactam and/or ceftazidime/avibactam may involve mutations leading to the structural modification of narrow spectrum OXA-2 and OXA-10 acquired oxacillinases (Fraile-Ribot et al., 2017a,b).

Besides β-lactamases, there is increasing evidence on the role of target modification in *P. aeruginosa* β-lactam resistance. Particularly noteworthy are the mutations in *ftsI*, encoding PBP3, an essential high molecular class B PBP with transpeptidase activity (Chen et al., 2016). Indeed, data from CF patients (Diaz Caballero et al., 2015; López-Causapé et al., 2017), epidemic high-risk clones (Cabot et al., 2016b; Del Barrio-Tofiño et al., 2017) as well as from *in vitro* studies (Cabot et al., 2016a) have recently shown that PBP3 is under strong mutational pressure, and that specific mutations in this PBP contribute to β-lactam resistance development. R504C/H and F533L mutations are likely those most commonly reported, and are located within the protein domains implicated in the formation and stabilization of the inactivating complex β-lactam-PBP3 (Han et al., 2010). Moreover, these specific mutations have been documented to emerge *in vivo* during chronic respiratory infection in CF patients (Diaz Caballero et al., 2015; López-Causapé et al., 2017) and upon meropenem (Cabot et al., 2016a) and aztreonam (Jorth et al., 2017) exposure *in vitro*. However, the precise contribution of PBP3 mutations to β-lactam resistance phenotypes needs to be further investigated using isogenic strains. Likewise, despite unique polymorphisms have also been detected in some clinical strains for other PBPs, their role in β-lactam resistance, if any, still needs to be experimentally addressed.

Other relevant components of the β-lactam mutational resistome are the porins and RND efflux pumps. Mutational inactivation of OprD is well-known to be the primary carbapenem resistance mechanisms in *P. aeruginosa* (Lister et al., 2009; Castanheira et al., 2014). OprD inactivation typically results from indels or nonsense mutations, including the Q142X mutation, characteristic of the widespread ST175 high-risk clone (Cabot et al., 2016b). Additionally, some amino acid substitutions have also been recently associated with OprD-driven resistance, particularly in the CF setting (Richardot et al., 2015). Finally, carbapenem resistance may also result from *oprD* repression caused by mutations in the MexEF-OprN efflux pump regulators (*mexS/T*) or the ParRS two-component system (Li et al., 2015). Overexpression of MexAB-OprM, caused by mutation of several genes involved in its regulation (*mexR*, *nalC*, or *nalD*) increases MICs of most β-lactams except imipenem, whereas overexpression of MexXY (*mexZ*, *parSR*, *amgS* mutations) is particularly involved in cefepime resistance (Li et al., 2015). Additionally, sequence variations in unique residues are detected in the genes coding for the efflux pump (Del Barrio-Tofiño et al., 2017; López-Causapé et al., 2017); however, their contribution to resistance profiles, if any, still needs to be further explored.

Finally, another potentially relevant mutational β-lactam resistance mechanism is the selection of large (>200 kb) deletions affecting specific parts of the chromosome (Cabot et al., 2016a). Although the basis of the conferred resistance phenotype still needs to be further clarified, these mutants can be recognized by the characteristic brown pigment (pyomelanine) caused by the deletion of one of the affected genes, *hmgA*, coding for a homogentisate-1,2- dioxygenase. This type of deletion has been documented in both, *in vitro* evolved β-lactam-resistant mutants and CF isolates (Cabot et al., 2016a; Hocquet et al., 2016). However, the deletion of *hmgA* is not responsible for the resistance phenotype, which could be linked to the deletion of another of the affected genes, *galU*. This gene codes for a UDP-glucose pyrophosphorylase involved in the synthesis of the lipopolysaccharide (LPS) core. Indeed, analysis of transposon mutant libraries has shown that inactivation of *galU* increases ceftazidime and meropenem MICs (Dötsch et al., 2009; Alvarez-Ortega et al., 2010).

## Aminoglycoside Mutational Resistome

In the absence of horizontally-acquired aminoglycoside modifying enzymes, resistance to this antibiotic class has been particularly linked to the mutational overexpression of MexXY-OprM. Indeed, mutational overexpression of this pump, mainly caused by *mexZ*, *amgS*, or *parRS* mutations (**Table 1**), is very frequent among clinical isolates, from both, CF patients and nosocomial infections (Guénard et al., 2014; Prickett et al., 2017). Moreover, recent studies show that the epidemic high-risk clone ST175 overexpresses MexXY due to a specific mutation in *mexZ* (G195E) (Cabot et al., 2016b). However, recent studies have revealed that the aminoglycoside mutational resistome extends far beyond MexXY overexpression, and that high-level resistance may result from the accumulation of multiple mutations, and the involvement of several novel resistance determinants has been recently documented (El’Garch et al., 2007; Schurek et al., 2008; Feng et al., 2016). Among them is particularly noteworthy *fusA1*, coding for the elongation factor G. Indeed, specific FusA1 mutations have been associated with aminoglycoside resistance *in vitro* (Feng et al., 2016; López-Causapé et al., 2018) and among clinical, particularly CF, strains (Chung et al., 2012; Markussen et al., 2014; Greipel et al., 2016; López-Causapé et al., 2017, 2018). Moreover, the implication of *fusA1* mutations in aminoglycoside resistance has been recently confirmed through site-directed mutagenesis (Bolard et al., 2017).

## Fluoroquinolone Mutational Resistome

The fluoroquinolone mutational resistome generally includes specific missense mutations in DNA gyrase (*gyrA* and/or *gyrB*) and topisomerase IV (*parC* and/or *parE*) Quinolone Resistance-Determining Regions (QRDRs) (**Table 1**) (Bruchmann et al., 2013; Kos et al., 2015). High-level fluoroquinolone resistance in *P. aeruginosa* high-risk clones is nearly universal, and typically involves combinations of mutations in GyrA-T83 and ParC-S87 (Del Barrio-Tofiño et al., 2017). QRDR mutations involved in fluoroquinolone resistance in CF might be more variable (López-Causapé et al., 2017). It is also well-known that the mutational overexpression of efflux pumps modulate fluoroquinolone resistance (**Table 1**). While the overexpression of MexAB-OprM and MexXY-OprM is globally more frequent among clinical strains, its contribution to clinical fluoroquinolone resistance is likely more modest (Bruchmann et al., 2013). On the other hand, the mutational overexpression of MexEF-OprN or MexCD-OprJ is associated with high-level (clinical) fluoroquinolone resistance, and although their prevalence is considered low except in the CF chronic infection setting, recent data show that it might be higher than expected (Richardot et al., 2015).

## Polymixin Mutational Resistome

Whereas the prevalence of polymyxin (colistin and polymyxin B) resistance is still globally low (<5%), it has increased in the last years because of the frequent use of these last-resource antibiotics for the treatment of MDR/XDR nosocomial and CF strains. Polymyxin resistance results most frequently from the modification of the LPS caused by the addition of a 4-amino-4-deoxy-L-arabinose moiety in the lipid A structure (Olaitan et al., 2014; Jeannot et al., 2017). The involved mutations are frequently located in the PmrAB or PhoPQ two-component regulators, which lead to the activation of the *arnBCADTEF* operon (Barrow and Kwon, 2009). More recent studies have revealed that mutations in the ParRS two-component regulator, not only produce polymyxin resistance due to the activation of the *arnBCADTEF* operon, but also lead to a MDR phenotype determined by the overprexpression of MexXY and the repression of OprD (Muller et al., 2011). Moreover, two additional two-component regulators, ColRS and CprRS, have been recently found to be involved in polymyxin resistance (Gutu et al., 2013). The analysis of colistin resistance mechanisms among clinical strains is not always straight forward, since the presence of mutations in these two-component regulators is not always associated with clinical colistin resistance, probably denoting partial complementation between the different regulators (Moskowitz et al., 2012; Gutu et al., 2013; López-Causapé et al., 2017). Moreover, recent *in vitro* evolution assays have revealed the implication of additional mutations in high level colistin resistance, facilitated by the emergence of mutator (*mutS* deficient) phenotypes (Dößelmann et al., 2017). Particularly noteworthy among them are those occurring in LptD, LpxC, or MigA.

## Other Antibiotics

Even if not considered a classical antipseudomonal agent, fosfomycin has emerged in the last decade as a potentially useful antibiotic in urinary tract infections and combined therapy for MDR/XDR *P. aeruginosa* (Michalopoulos et al., 2011). However, fosfomycin resistance spontaneous mutation rates are high and the mechanism involved is typically the mutational inactivation of *glpT*, coding for a glycerol-3-phospate permease required for fosfomycin uptake (Castañeda-García et al., 2009; Rodríguez-Rojas et al., 2010). *glpT* mutations, conferring high-level fosfomycin resistance are also frequently found among MDR/XDR high-risk clones (Del Barrio-Tofiño et al., 2017), and some specific mutations, such as T211P, have been fixed in some widespread lineages as described for ST175 (Cabot et al., 2016b). Another potentially useful antimicrobial for combined therapy against MDR/XDR *P. aeruginosa* is rifampicin (Cai et al., 2017). However, rifampicin resistance may emerge at high frequency due to the selection of specific missense mutations in *rpoB*, coding for the beta subunit of the RNA polymerase (Jatsenko et al., 2010). Another example of newer antibiotic families with antipseudomonal activity are the pacidamycins, uridyl peptide antibiotics, targeting translocase I, an essential enzyme in peptidoglycan biosynthesis (Mistry et al., 2013). Emergence of high-level resistance to this antibiotic class has been shown to involve the selection of mutations in the Opp transporter, a binding protein-dependent ABC transporter used for oligopeptide import. Finally, the *P. aeruginosa* mutational resistome, particularly in the CF setting, may also include resistance to other used antibiotics such as the frequent mutations of domain V of 23S rRNA, conferring macrolide resistance (Mustafa et al., 2017).

## Concluding Remarks and Future Perspectives

The analysis of the *P. aeruginosa* mutational resistome, together with the analysis of the horizontally-acquired resistance determinants, should be useful for establishing the antibiotic resistance genotype, which should correlate with the antibiotic resistance phenotype and as such, it should permit the design of therapeutic strategies and for monitoring the efficacy of administered antibiotic treatments. However, the current applicability of the analysis of the mutational resistome is still limited by the large number of genes involved and the complexity of the resistance phenotypes generated, and, particularly, by the difficulties, in many cases, for differentiating relevant mutations from simple natural polymorphisms. Obviously, the evolution of the mutational resistome is a direct consequence of antimicrobial exposure and as such, it is not surprising that exposure to one antibiotic drives evolution of the mutational resistome for that antibiotic. However, the complexity of the actual resistance profile is further increased by the specificity and interactions among different resistance mechanisms. Indeed, a resistance mutation selected by one antibiotic may have a variable effect among the different agents within the same antibiotic class or family. Likewise, cross resistance (or collateral resistance) implies that exposure to one antibiotic drives also the development of resistance to a different one from the same or other classes. Typically, this is caused by the developed resistance mechanism (such as efflux pump overexpression) affecting simultaneously different antibiotics. Indeed, potential development of cross resistance is a major issue to consider when using antibiotic combinations (Vestergaard et al., 2016). Moreover, cross resistance between antibiotics and antiseptics and other biocides may also occur (Li et al., 2015). Perhaps less obvious is collateral susceptibility, which implies that exposure to one antibiotic increases the susceptibility to a different one (Pál et al., 2015; Imamovic et al., 2017). This might be achieved through two mechanisms. One possible mechanism is that exposure to one antibiotic directly causes increased susceptibility to a different one, for example, mutations in the β-lactamase AmpC increases cephalosporin hydrolysis while reducing that of penicillins or carbapenems (Cabot et al., 2014). The second possibility is that the development of a resistance mechanism impairs the activity of another existing resistance mechanism. An example is competition between the different efflux pumps, since the overexpression of one may impair the expression of another (Mulet et al., 2011). Thus, the evolution of the mutational resistome for a given antibiotic is not only dependent on the exposure to this antibiotic, but it is also conditioned by the simultaneous or even previous exposures to other antibiotics. An illustrative example is provided in a recent *in vitro* study that demonstrated, for a broad range of antibiotic classes, that the history of exposure and resistance development to a given antibiotic, conditions the dynamics and mechanisms of resistance development when exposed to a second one (Yen and Papin, 2017). In summary, the comprehensive analysis of the mutational resistome of *P. aeruginosa* in CF and nosocomial infections is expected to become a useful tool for optimizing therapeutic strategies and monitoring the efficacy of administered antibiotic treatments in the near future.

## Author Contributions

CL-C and AO wrote the manuscript. CL-C, GC, EdB-T, and AO reviewed the literature.

## Conflict of Interest Statement

The authors declare that the research was conducted in the absence of any commercial or financial relationships that could be construed as a potential conflict of interest.
